# Systems biological assessment of immunity to mild versus severe COVID-19 infection in humans

**DOI:** 10.1126/science.abc6261

**Published:** 2020-08-11

**Authors:** Prabhu S. Arunachalam, Florian Wimmers, Chris Ka Pun Mok, Ranawaka A. P. M. Perera, Madeleine Scott, Thomas Hagan, Natalia Sigal, Yupeng Feng, Laurel Bristow, Owen Tak-Yin Tsang, Dhananjay Wagh, John Coller, Kathryn L. Pellegrini, Dmitri Kazmin, Ghina Alaaeddine, Wai Shing Leung, Jacky Man Chun Chan, Thomas Shiu Hong Chik, Chris Yau Chung Choi, Christopher Huerta, Michele Paine McCullough, Huibin Lv, Evan Anderson, Srilatha Edupuganti, Amit A. Upadhyay, Steve E. Bosinger, Holden Terry Maecker, Purvesh Khatri, Nadine Rouphael, Malik Peiris, Bali Pulendran

**Affiliations:** 1 Institute for Immunity, Transplantation and Infection, Stanford University School of Medicine, Stanford, CA 94305, USA.; 2 HKU-Pasteur Research Pole, School of Public Health, HKU Li Ka Shing Faculty of Medicine, The University of Hong Kong (HKU), Hong Kong.; 3 Centre of Influenza Research, School of Public Health, HKU Li Ka Shing Faculty of Medicine, HKU, Hong Kong.; 4 Center for Biomedical Informatics, Department of Medicine, Stanford University School of Medicine, Stanford, CA 94305, USA.; 5 Hope Clinic of the Emory Vaccine Center, Department of Medicine, Division of Infectious Diseases, Emory University School of Medicine, Decatur, GA 30030, USA.; 6 Infectious Diseases Centre, Princess Margaret Hospital, Hospital Authority of Hong Kong, Hong Kong.; 7 Stanford Functional Genomics Facility, Stanford University School of Medicine, Stanford, CA 94305, USA.; 8 Emory Vaccine Center, Yerkes National Primate Research Center, Atlanta, GA 30329, USA.; 9 Department of Pediatrics, Division of Infectious Disease, Emory University School of Medicine, Atlanta, GA 30322, USA.; 10 Department of Pathology and Laboratory Medicine, Emory University, Atlanta, GA 30329, USA.; 11 Department of Pathology, Stanford University School of Medicine, Stanford, CA 94305, USA.; 12 Department of Microbiology and Immunology, Stanford University School of Medicine, Stanford, CA 94305, USA.

## Abstract

Coronavirus disease 2019 (COVID-19) represents a global crisis, yet major knowledge gaps remain about human immunity to severe acute respiratory syndrome coronavirus 2 (SARS-CoV-2). We analyzed immune responses in 76 COVID-19 patients and 69 healthy individuals from Hong Kong and Atlanta, Georgia, United States. In the peripheral blood mononuclear cells (PBMCs) of COVID-19 patients, we observed reduced expression of human leukocyte antigen class DR (HLA-DR) and proinflammatory cytokines by myeloid cells as well as impaired mammalian target of rapamycin (mTOR) signaling and interferon-α (IFN-α) production by plasmacytoid dendritic cells. By contrast, we detected enhanced plasma levels of inflammatory mediators—including EN-RAGE, TNFSF14, and oncostatin M—which correlated with disease severity and increased bacterial products in plasma. Single-cell transcriptomics revealed a lack of type I IFNs, reduced HLA-DR in the myeloid cells of patients with severe COVID-19, and transient expression of IFN-stimulated genes. This was consistent with bulk PBMC transcriptomics and transient, low IFN-α levels in plasma during infection. These results reveal mechanisms and potential therapeutic targets for COVID-19.

## Immune profiling of COVID-19 patients

Coronavirus disease 2019 (COVID-19) has affected millions of people globally, yet how the human immune system responds to and influences COVID-19 severity remains unclear. Mathew *et al.* present a comprehensive atlas of immune modulation associated with COVID-19. They performed high-dimensional flow cytometry of hospitalized COVID-19 patients and found three prominent and distinct immunotypes that are related to disease severity and clinical parameters. Arunachalam *et al.* report a systems biology approach to assess the immune system of COVID-19 patients with mild-to-severe disease. These studies provide a compendium of immune cell information and roadmaps for potential therapeutic interventions.

*Science*, this issue p. eabc8511, p. 1210

## Teaser

Immune responses of COVID-19 patients are cataloged and compared with those of healthy individuals.

The recent emergence of the severe acute respiratory syndrome coronavirus 2 (SARS-CoV-2) in Wuhan, China, in December 2019 and its rapid international spread caused a global pandemic. Research has moved rapidly in isolating, sequencing, and cloning the virus; developing diagnostic kits; and testing candidate vaccines. However, key questions remain about the dynamic interaction between the human immune system and the SARS-CoV-2 virus.

Coronavirus disease 2019 (COVID-19) presents with a spectrum of clinical phenotypes, with most patients exhibiting mild to moderate symptoms and 15% of patients progressing, typically within a week, to severe or critical disease that requires hospitalization (*1*). A minority of those who are hospitalized develop acute respiratory disease syndrome (ARDS) and require mechanical ventilation. Epidemiological data so far suggest that COVID-19 has a case fatality rate several times greater than that of seasonal influenza (*1*). The elderly and individuals with underlying medical comorbidities such as cardiovascular disease, diabetes mellitus, chronic lung disease, chronic kidney disease, obesity, hypertension, or cancer have a much higher mortality rate than healthy young adults (*2*). The underlying causes of this difference are unknown, but they may be due to an impaired interferon (IFN) response and dysregulated inflammatory responses, as have been observed with other zoonotic coronavirus infections such as severe acute respiratory syndrome (SARS) and Middle East respiratory syndrome (MERS) (*3*). Current research is uncovering how the adaptive immune response to SARS-CoV-2 is induced with optimal functional capacities to clear SARS-CoV-2 viral infection (*4*–*6*).

Understanding the immunological mechanisms underlying the diverse clinical presentations of COVID-19 is a crucial step in the design of rational therapeutic strategies. Recent studies have suggested that COVID-19 patients are characterized by lymphopenia and increased numbers of neutrophils (*7*–*9*). Most patients with severe COVID-19 exhibit enhanced levels of proinflammatory cytokines including interleukin-6 (IL-6) and IL-1β as well as MCP-1, IP-10, and granulocyte colony-stimulating factor (G-CSF) in the plasma (*10*). It has been proposed that high levels of proinflammatory cytokines might lead to shock as well as respiratory failure or multiple organ failure, and several trials to assess inflammatory mediators are under way (*11*). However, little is known about the immunological mechanisms underlying COVID-19 severity and the extent to which they differ from the immune responses to other respiratory viruses. Furthermore, the question of whether individuals in different parts of the world respond differently to SARS-CoV-2 remains unknown. In this study, we used a systems biological approach [mass cytometry and single-cell transcriptomics of leukocytes, transcriptomics of bulk peripheral blood mononuclear cells (PBMCs), and multiplex analysis of cytokines in plasma] to analyze the immune response in 76 COVID-19 patients and 69 age- and sex-matched controls from two geographically distant cohorts.

## Analysis of peripheral blood leukocytes from COVID-19 patients by mass cytometry

**Fig. 1:**
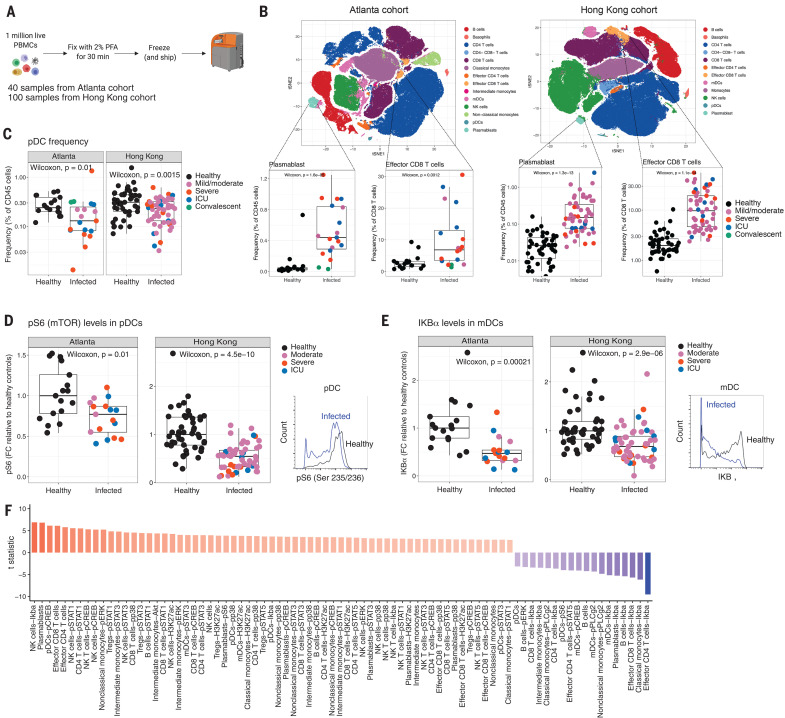
Mass cytometry analysis of human peripheral blood leukocytes from COVID-19 patients. (**A**) A schematic representation of the experimental strategy. PFA, paraformaldehyde. (**B**) Representation of mass cytometry–identified cell clusters visualized by t-SNE in two-dimensional space. The box plots on the bottom show frequency of plasmablasts (CD3^−^, CD20^−^, CD56^−^, HLA-DR^+^, CD14^−^, CD16^−^, CD11c^−^, CD123^−^, CD19^lo^, CD27^hi^, and CD38^hi^) and effector CD8 T cells (CD3^+^, CD8^+^, CD38^hi^, and HLA-DR^hi^) in both cohorts. (**C**) Frequencies of pDCs (CD3^−^, CD20^−^, CD56^−^, HLA-DR^+^, CD14^−^, CD16^−^, CD11c^−^, and CD123^+^) in healthy and COVID-19–infected individuals in both cohorts. (**D** and **E**) Box plots showing fold change (FC) of pS6 staining in pDCs (D) and IκBα staining in mDCs (E) relative to the medians of healthy controls. The histograms on the right depict representative staining of the same. (**F**) Distinguishing features [false discovery rate (FDR) < 0.01] through linear modeling analysis of the mass cytometry data between healthy and infected subjects. In all box plots, the boxes show median, upper, and lower quartiles. The whiskers show 5th to 95th percentiles. Each dot represents an individual sample (healthy: *n* = 17 and 45; infected: *n* = 19 and 54, for Atlanta and Hong Kong cohorts, respectively). For the t-SNE analysis, *n* = 34 and 60 for Atlanta and Hong Kong cohorts, respectively. The colors of the dots indicate the severity of clinical disease, as shown in the legends. The differences between the groups were measured by Mann-Whitney rank sum test (Wilcoxon, paired = FALSE). The *P* values depicting significance are shown within the box plots.

COVID-19–infected patient samples and samples from age- and sex-matched healthy controls were obtained from two independent cohorts: (i) the Princess Margaret Hospital at Hong Kong University and (ii) the Hope Clinic at Emory University in Atlanta, Georgia, United States. Patient characteristics and the different assays performed are shown in Table 1. We used mass cytometry to assess immune responses to SARS-CoV-2 infection in 52 COVID-19 patients, who were confirmed positive for viral RNA by polymerase chain reaction (PCR), and 62 age- and gender-matched healthy controls distributed between the two cohorts. To characterize immune cell phenotypes in PBMCs, we used a phospho-CyTOF panel that includes 22 cell surface markers and 12 intracellular markers against an assortment of kinases and phospho-specific epitopes of signaling molecules and H3K27ac—a marker of histone modification that drives epigenetic remodeling (*12*, *13*) (table S1). The experimental strategy is described in Fig. 1A. The phospho-CyTOF identified 12 main subtypes of innate and adaptive immune cells in both cohorts, as represented in the t-distributed stochastic neighbor embedding (t-SNE) plots (Fig. 1B). There was a notable increase in the frequency of plasmablast and effector CD8 T cells in all infected individuals (Fig. 1B) in both cohorts, as has been described recently in other studies (*6*, *8*, *14*). Of note, the kinetics of the CD8 effector T cell response were prolonged and continued to increase up to day 40 after onset of the symptoms (fig. S1).

**Table 1:** Patient characteristics and number of samples used in different assays. NA, not applicable.

**Characteristics**	**Hong Kong cohort**	**Atlanta cohort**
*Number of subjects*		
COVID-19	36 patients*	40 patients*
Flu/RSV	NA	16 patients
Healthy	45 individuals	24 individuals
*Age*		
COVID-19 [median (range)]	55 (18–80)	56 (25–94)
Flu/RSV	NA	66 (51–86)
Healthy	53 (21–69)	52 (23–91)
*Gender*		
COVID-19 (male, %)	58%	55%
Flu/RSV (male, %)	NA	31%
Healthy (male, %)	58%	42%
*Clinical severity of COVID-19 patients*		
Mild/moderate	75%	18%
Severe (no ICU)	14%	60%
ICU	11%	18%
*Clinical severity of flu/RSV patients*		
Mild/moderate	NA	37.5%
Severe (no ICU)	NA	37.5%
ICU	NA	31%
*Intervention*		
IFN-β1	20%	NA
Corticosteroids	19%	NA
Antivirals	61%	NA
*Assays using COVID-19 samples*		
Phospho-CyTOF	54 PBMC samples (36 patients)	19 PBMC samples (16 patients)
In vitro stimulation	NA	17 PBMC samples (15 patients)
Olink proteomics	NA	36 plasma samples (29 patients)
CITE-seq	NA	7 PBMC samples (7 patients)
Bulk RNA-seq	NA	17 PBMC samples (15 patients)
Bacterial products	NA	51 plasma samples (40 patients)
*Assays using flu/RSV samples*		
Phospho-CyTOF	NA	4 PBMC samples (4 patients)
Olink proteomics	NA	19 plasma samples (16 patients)

*Some patients have blood from multiple time points.

We next used manual gating to identify 25 immune cell subsets (fig. S2) and determined whether there were changes in the frequency or signaling molecules of innate immune cell populations consistent between the two cohorts. There were several differences, but notably the frequency of plasmacytoid dendritic cells (pDCs) was significantly reduced in the PBMCs of SARS-CoV-2–infected individuals in both cohorts (Fig. 1C). The kinetics of pDC response did not show an association with the time since symptom onset (fig. S1C). Neither did the observed changes correlate with the clinical severity of infection (fig. S1). Additionally, there was reduced expression of pS6 [(phosphorylated ribosomal protein S6), a canonical target of mammalian target of rapamycin (mTOR) activation (*15*)] in pDCs and decreased IκBα—an inhibitor of the signaling of the NF-κβ transcription factor—in myeloid dendritic cells (mDCs) (Fig. 1, D and E). mTOR signaling is known to mediate the production of interferon-α (IFN-α) in pDCs (*16*), which suggests that pDCs may be impaired in their capacity to produce IFN-α in COVID-19 patients. Finally, we used a linear modeling approach to detect features that distinguish healthy from infected individuals and those that discriminate individuals on the basis of the clinical severity of COVID-19. This analysis was performed with the cohort (Hong Kong or Atlanta) as a covariate to identify only features that were consistent across both cohorts. The distinguishing features between healthy and infected individuals are shown in Fig. 1F. These include frequencies of plasmablast and effector T cells and the changes in innate immune cells described above in addition to STAT1 (signal transducer and activator of transcription 1) and other signaling events in T cells and natural killer (NK) cells. Of note, no features were significantly different between clinical severity groups.

We further examined the effect of various therapeutic interventions on the immune responses using samples from the Hong Kong cohort, in which some patients were treated with IFN-β1, corticosteroids, or antivirals. The infected individuals, irrespective of the intervention, showed an increased plasmablast and effector CD8 T cell frequency compared with healthy controls (fig. S3). However, there was an increased frequency of effector CD8 T cells (fig. S3, bottom panel, right column) and decreased pS6 signal in the pDCs of antiviral-treated individuals (fig. S4).

## COVID-19 results in functional impairment of blood myeloid cells and pDCs

**Fig. 2:**
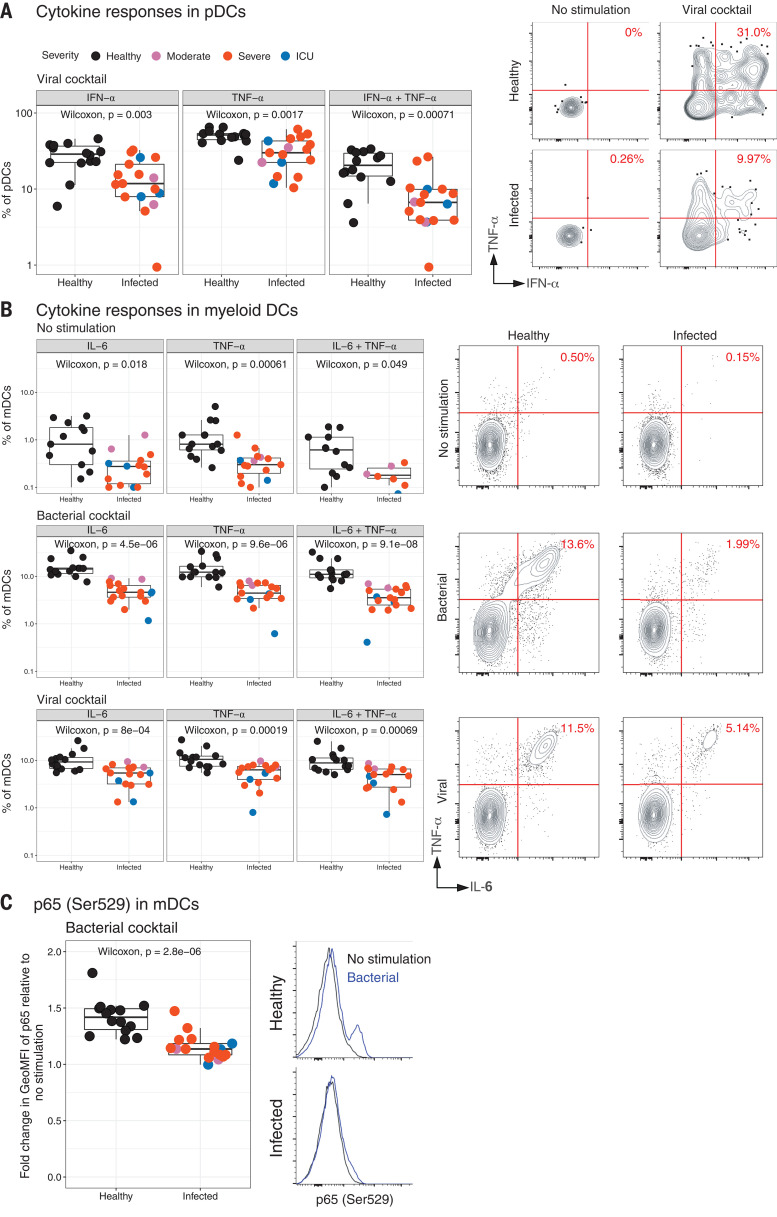
Flow cytometry analysis of ex vivo stimulated human peripheral blood leukocytes from COVID-19 patients. (**A**) Box plots showing the fraction of pDCs in PBMCs of healthy or infected donors (CD3^−^, CD20^−^, CD56^−^, HLA-DR^+^, CD14^−^, CD16^−^, CD11c^−^, and CD123^+^) producing IFN-α, TNF-α, or IFN-α + TNF-α in response to stimulation with the viral cocktail (polyIC + R848). The contour plots on the right show IFN-α, TNF-α, or IFN-α + TNF-α staining in pDCs. (**B**) Box plots showing the fraction of mDCs in PBMCs of healthy or infected donors (CD3^−^, CD20^−^, CD56^−^, HLA-DR^+^, CD14^−^, CD16^−^, CD123^+^, and CD11c^−^) producing IL-6, TNF-α, or IL-6 + TNF-α in response to no stimulation (top), the bacterial cocktail (middle; Pam3CSK4, LPS, and Flagellin), or the viral cocktail (bottom; polyIC + R848). The flow cytometry plots on the right are representative plots gated on mDCs showing IL-6, TNF-α, or IL-6 + TNF-α response. (**C**) Fold change of NF-κβ p65 (Ser^529^) staining in PBMCs stimulated with bacterial cocktail relative to no stimulation in healthy and infected donors to show the reduced induction of p65 phosphorylation in infected individuals. The histograms show representative flow cytometry plots of p65 staining in mDCs. GeoMFI, geometric mean fluorescence intensity. In all box plots, the boxes show median, upper, and lower quartiles. The whiskers show 5th to 95th percentiles. Each dot represents an Atlanta cohort patient (*n* = 14 and 17 for healthy and infected, respectively). Colors of the dots indicate the severity of clinical disease, as shown in the legends. The differences between the groups were measured by Mann-Whitney rank sum test. The *P* values depicting significance are shown within the box plots.

Given the earlier findings that mTOR signaling in pDCs mediates the production of IFN-α in response to Toll-like receptor (TLR) stimulation (*16*), the reduced expression of pS6 in pDCs suggests that such cells may be impaired in their capacity to produce IFN-α. To test this, we performed ex vivo stimulation of PBMCs from healthy or COVID-19–infected individuals, using a mixture of synthetic TLR7 and TLR 8 (TLR7/8) and TLR3 ligands, which are known to be expressed by viruses, and we performed an intracellular staining assay to detect cytokine responses. The TLR ligands included TLR3 and TLR7/8 ligands, polyIC and R848. Consistent with our hypothesis, there was reduced production of IFN-α in response to the TLR stimuli in the pDCs of infected individuals compared with those of healthy controls (Fig. 2A). The TNF-α response was also significantly reduced in the pDCs of infected individuals, which demonstrates that the pDCs are functionally impaired in COVID-19 infection. We also determined the ability of mDCs and CD14^+^ monocytes to respond to TLR stimuli. Notably, the response in mDCs as well as that in monocytes were also significantly lower in response to stimulation with a bacterial ligand cocktail (composed of TLR2, TLR4, and TLR5 ligands) or with the viral TLR cocktail (Fig. 2B and fig. S5). Furthermore, the reduced IκBα levels did not translate into enhanced NF-κβ subunit p65 phosphorylation as measured by p65 (Ser^529^) in the same cells (Fig. 2C). These results suggest that the innate immune cells in the periphery of COVID-19–infected individuals are suppressed in their response to TLR stimulation, irrespective of the clinical severity.

## Enhanced concentrations of cytokines and inflammatory mediators in plasma from COVID-19 patients

**Fig. 3:**
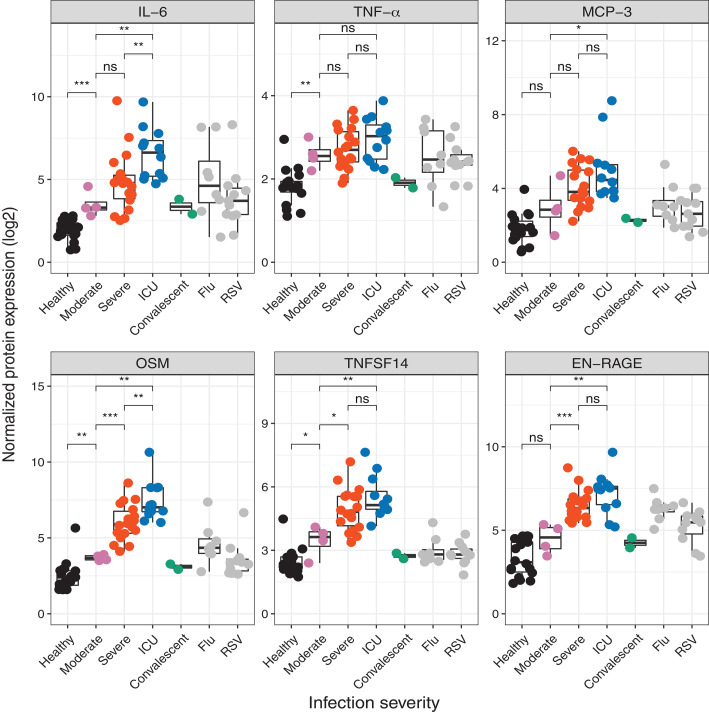
Multiplex analysis of cytokines in the plasma of COVID-19 patients. Cytokine levels in the plasma of healthy or infected individuals. The infected individuals are further classified on the basis of the severity of their clinical COVID-19 disease. The normalized protein expression values plotted on the *y* axes are arbitrary units defined by Olink Proteomics to represent Olink data. In all box plots, the boxes show median, upper, and lower quartiles. The whiskers show 5th to 95th percentiles. Each dot represents an Atlanta cohort sample (*n* = 18 healthy, 4 moderate, 18 severe, 12 ICU, 2 convalescent, 8 flu, and 11 RSV). The colors of the dots indicate the severity of clinical disease, as shown in the legends. The differences between the groups were measured by Mann-Whitney rank sum test (Wilcoxon, paired = FALSE; *P < 0.05; **P < 0.01; ***P < 0.001; ns, not significant).

The impaired cytokine response of myeloid cells and pDCs in response to TLR stimulation was unexpected and seemingly at odds with the literature describing an enhanced inflammatory response in COVID-19–infected individuals. Several studies have described higher plasma levels of cytokines, including but not limited to IL-6, TNF-α, and CXCL10 (*10*, *17*–*19*). Therefore, we evaluated cytokines and chemokines in plasma samples from the Atlanta cohort using the Olink multiplex inflammation panel that measures 92 different cytokines and chemokines. Of the 92 analytes measured, 71 proteins were detected within the dynamic range of the assay. Of these 71 proteins, 43 cytokines, including IL-6, MCP-3, and CXCL10, were significantly up-regulated in COVID-19 infection (Fig. 3, top row, and fig. S6). These results demonstrate that plasma levels of inflammatory molecules were significantly up-regulated, despite the impaired cytokine response in blood myeloid cells and pDCs, which suggests a tissue origin of the plasma cytokines.

In addition to IL-6 and other cytokines described previously (*10*), we identified three proteins that were significantly enhanced in COVID-19 infection and strongly correlated with clinical severity (Fig. 3, bottom row). These were TNFSF14 [LIGHT, a ligand of lymphotoxin B receptor that is highly expressed in human lung fibroblasts and implicated in lung tissue fibrosis and remodeling and inflammation (*20*)], EN-RAGE [S100A12, a biomarker of pulmonary injury that is implicated in pathogenesis of sepsis-induced ARDS (*21*)], and oncostatin M [(OSM), a regulator of IL-6]. Of note, the TNFSF14 is distinctively enhanced in the plasma of COVID-19–infected individuals but not in cases of other related pulmonary infections such as influenza (flu) virus and respiratory syncytial virus (RSV) (Fig. 3). Given the pronounced and unappreciated observations of the enhanced plasma concentrations of TNFSF14, EN-RAGE, and OSM and their correlation to disease severity, we used an enzyme-linked immunosorbent assay (ELISA) to independently validate these results. Consistent with the multiplex Olink analysis, we found a significant increase of these inflammatory mediators in the plasma of severe and intensive care unit (ICU) COVID-19 patients. Furthermore, we found a correlation between multiplex analysis by Olink and the ELISA results (fig. S7). These results suggest that COVID-19 infection induces a distinctive inflammatory program characterized by cytokines released from tissues (most likely the lungs) but suppression of the innate immune system in the periphery. These observations may also represent previously unexplored therapeutic strategies for intervention against severe COVID-19.

## Single-cell transcriptional response to COVID-19 infection

**Fig. 4:**
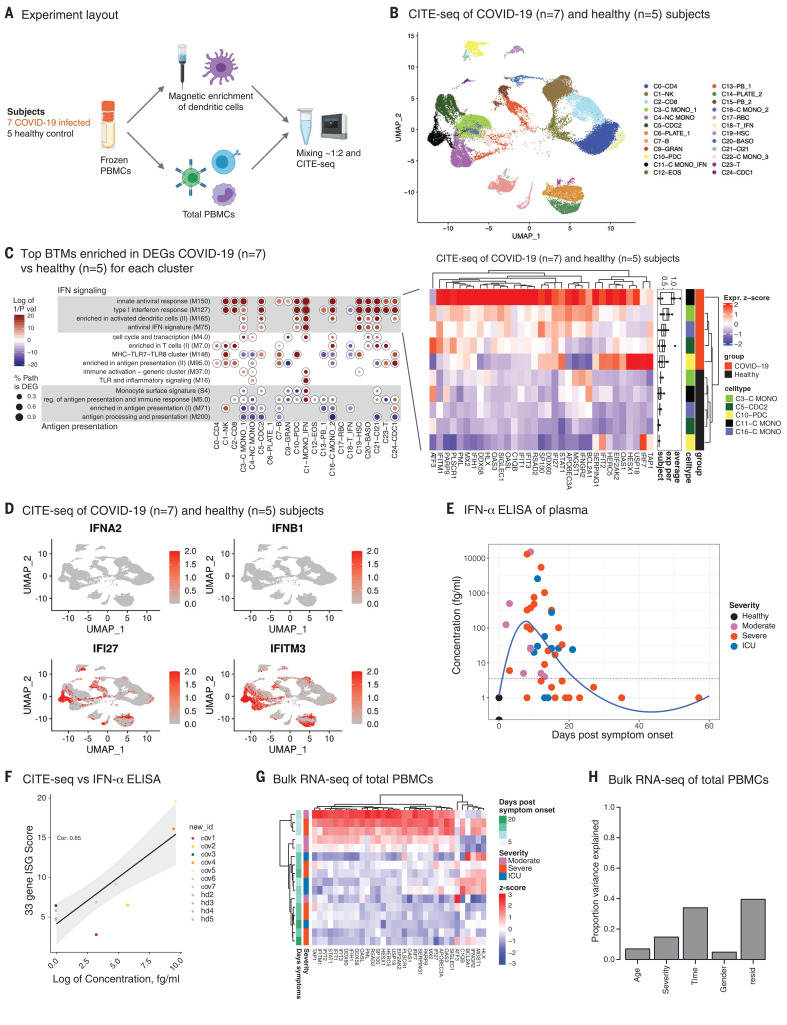
Early, transient ISG expression in COVID-19 infection. (**A**) A schematic representation of the DC enrichment strategy used in CITE-seq analysis. (**B**) UMAP representation of PBMCs from all analyzed samples (*n* = 12), colored by manually annotated cell type. (**C**) Pairwise comparison of genes from healthy individuals (*n* = 5) and COVID-19–infected patients (*n* = 7) was conducted for each cluster. DEGs were analyzed for overrepresentation of BTMs. The ringplot shows overrepresented pathways in up- and down-regulated genes of each cluster. The heatmap on the right shows the average expression levels of 33 ISGs derived from the enriched BTMs in different cell clusters of healthy (*n* = 5) and COVID-19 subjects (*n* = 7). (**D**) UMAP representation of PBMCs from all analyzed samples showing the expression levels of selected IFNs and ISGs. (**E**) Kinetics of circulating IFN-α levels (femtograms per milliliter) in plasma measured using SIMoA technology (*n* = 18 healthy and 40 COVID-19–infected patients). (**F**) Correlation between circulating IFN-α levels in plasma and the average expression of ISGs measured by CITE-seq analysis. (**G**) Hierarchically clustered heatmap of the expression of the CITE-seq ISG signature (C) in the bulk RNA-seq dataset, performed using an extended group of subjects (*n* = 17 healthy and 17 COVID-19–infected samples). Colors represent gene-wise *z* scores. (**H**) Bar chart representing the proportion of variance in CITE-seq ISG signature expression explained by the covariates in the *x* axis through principal variance component analysis (PVCA). resid, residual. (figure on next page)

To investigate the molecular and cellular processes that lead to the distinctive inflammatory program, we used cellular indexing of transcriptomes and epitopes by sequencing (CITE-seq) and profiled the gene and protein expression in PBMC samples of COVID-19–infected individuals. Cryopreserved PBMC samples from a total of 12 age-matched subjects in the Atlanta cohort (five healthy controls and seven COVID-19 patients; Table 2) were enriched for DCs, stained using a cocktail of 36 DNA-labeled antibodies (table S2), and analyzed using droplet-based single-cell gene expression profiling approaches (Fig. 4A). We performed the experiment in two batches and obtained transcriptomes for more than 63,000 cells after initial preprocessing. Next, we generated a cell-by-gene matrix and conducted dimensionality reduction through uniform manifold approximation and projection (UMAP) and graph-based clustering. Analysis of cell distribution within the UMAP between experiments revealed no major differences, and we analyzed the datasets from the two experiments together without batch correction (fig. S8). Next, we calculated the per-cell quality control (QC) metrics (fig. S9), differentially expressed genes (DEGs) in each cluster compared with all other cells (fig. S10 and table S4), and the abundance of DNA-labeled antibodies in each cell (fig. S11). Using this information, we filtered low-quality cells and manually annotated the clusters. After QC and cluster annotation, we retained a final dataset with 57,669 high-quality transcriptomes and a median of ~4781 cells per sample and 1803 individual genes per cell that we used to construct the single-cell immune cell landscape of COVID-19 (Fig. 4B).

**Table 2:** Detailed characteristics of patient samples used in the CITE-seq analysis. Dashes indicate that the information is not applicable. dec., deceased; F, female; M, male; B, Black; W, white.

**ID**	**Infection**	**Response**	**ICU**	**Day**	**Age**	**Sex**	**Ethnicity**
cov1	COVID-19	Severe, dec.	Y	15	60	F	B
cov2	COVID-19	Severe	N	15	75	F	W
cov3	COVID-19	Severe	N	16	59	M	B
cov4	COVID-19	Severe	N	8	48	M	B
cov5	COVID-19	Moderate	N	9	53	F	B
cov6	COVID-19	Moderate	N	2	75	F	W
cov7	COVID-19	Moderate	N	9	47	F	B
hd1	Healthy	–	–	–	84	F	W
hd2	Healthy	–	–	–	68	F	W
hd3	Healthy	–	–	–	38	M	W
hd4	Healthy	–	–	–	90	M	W
hd5	Healthy	–	–	–	70	F	W

We observed several clusters that were primarily identified in COVID-19–infected individuals, including a population of plasmablasts, platelets, and red blood cells and several populations of granulocytes. Notably, we detected clusters of T cells and monocytes that were characterized by the expression of interferon-stimulated genes (ISGs) such as IFI27, IFITM3, or ISG15 (see C11-C MONO_IFN and C18-T_IFN in fig. S10). These IFN response–enriched clusters emerged only in samples from COVID-19 patients (fig. S12).

To describe the specific transcriptional state of single cells from COVID-19–infected individuals, we determined the DEGs for cells from all COVID-19–infected samples in a given cluster compared with the cells from all healthy individuals in the same cluster. We then analyzed these DEGs with overrepresentation analysis using blood transcriptional modules (BTMs) (*22*) to better understand which immune pathways are differentially regulated in patients with COVID-19 compared with healthy individuals (Fig. 4C and fig. S13). The analysis indicated a marked induction of antiviral BTMs, especially in cell types belonging to the myeloid and dendritic cell lineage. Detailed analysis of the expression pattern of the distinct union of genes driving the enrichment of these antiviral pathways in monocytes and dendritic cells revealed that many ISGs were up-regulated in these cell types (Fig. 4C, heatmap). Given our observations of muted IFN-α production in pDCs (Fig. 2A), we investigated the expression of genes encoding various type I and type II IFNs across cell types (Fig. 4D and fig. S14). Notably, with the exception of modest levels of IFN-γ expression in T and NK cells, we could not detect any expression of IFN-α and -β genes, which is consistent with the functional data demonstrating impaired type I IFN production by pDCs and myeloid cells (Fig. 2). However, there was an enhanced expression of ISGs in patients with COVID-19 (Fig. 4D) in spite of an impaired capacity of the innate cells in the blood compartment to produce these cytokines.

Despite the lack of type I IFN gene expression, the presence of an ISG signature in the PBMCs of COVID-19–infected individuals raised the possibility that low quantities of type I IFNs produced in the lung by SARS-CoV-2 infection (*17*) might circulate in the plasma and induce the expression of ISGs in PBMCs. We thus measured the concentration of IFN-α in plasma using a highly sensitive ELISA enabled by single molecule array (SIMoA) technology. We observed a marked increase in the concentration of IFN-α, which peaked around day 8 after onset of symptoms and regressed to baseline levels by day 20 (Fig. 4E). Notably, we observed a strong correlation between the average expression levels of the ISG signature in PBMCs identified by CITE-seq analysis and the IFN-α concentration in plasma (Fig. 4F). Additionally, we noticed a strong temporal dependence of the IFN-α response.

To investigate this further and to independently validate the observations in the CITE-seq analysis, we performed bulk RNA sequencing (RNA-seq) analysis of PBMCs in an extended group of subjects (17 COVID-19 patients and 17 healthy controls) from the same cohort. We first evaluated whether the ISG signature containing 33 genes identified in the CITE-seq data was also observed in the bulk RNA-seq dataset. We observed a strong induction of the ISGs in COVID-19 subjects compared with healthy donors in this dataset as well (Fig. 4G). Of note, we did not detect expression of genes encoding IFN-α or IFN-β, consistent with the CITE-seq and flow cytometry experiments (Fig. 4D and Fig. 2, respectively). We also performed an unbiased analysis of an extended set of genes in the IFN transcriptional network (*23*) and found that these were induced in COVID-19 subjects relative to healthy controls, as observed for the limited ISG signature (fig. S15A). Similar to the observation with CITE-seq data (Fig. 4F), there was a strong correlation between circulating IFN-α and the ISG response measured by the bulk transcriptomics (fig. S15B). Additionally, we analyzed the individual impact of major covariates—time, disease severity, sex, and age—on the observed ISG signature. Although time emerged as the primary driver of ISG signature, COVID-19 clinical severity also had an effect (Fig. 4H and fig. S15C). Taken together, these data demonstrate that, early during SARS-CoV-2 infection, there are low levels of circulating IFN-α that induce ISGs in the periphery while the innate immune cells in the periphery are impaired in their capacity to produce inflammatory cytokines.

**Fig. 5:**
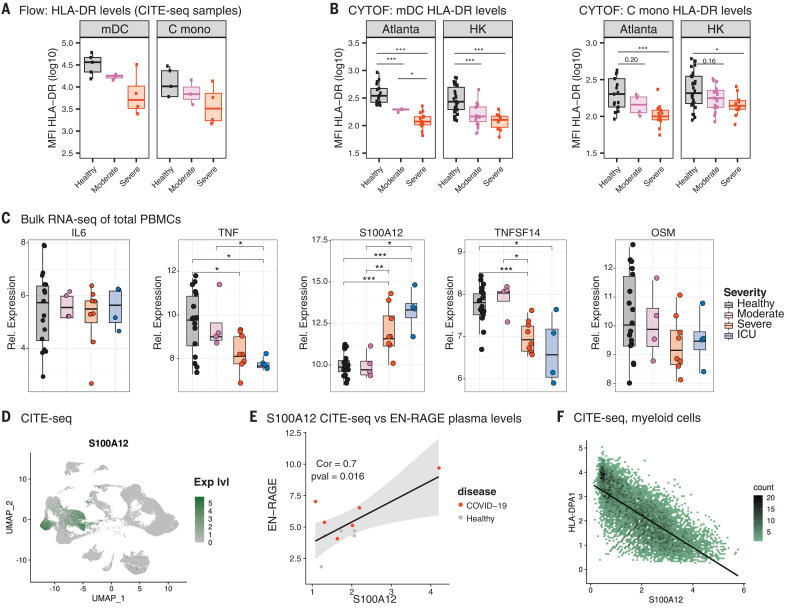
Attenuated inflammatory response in peripheral innate immune cells from COVID-19 patients. (**A**) Flow cytometry analysis of PBMCs analyzed in parallel to the CITE-seq experiment. The log_10_ median fluorescence intensity (MFI) of HLA-DR expression is shown. (**B**) Median intensity of HLA-DR expression in the phospho-CyTOF experiment from Fig. 1. Squares represent individual samples [Hong Kong (HK): healthy = 30, moderate = 15, and severe = 10; and Atlanta: healthy = 17, moderate = 4, and severe = 13]. The boxes indicate median, upper, and lower quartiles. The whisker length equals 1.5 times the interquartile range. (**C**) Relative (Rel.) expression of genes encoding different cytokines in the bulk RNA-seq dataset. The boxes show median, upper, and lower quartiles, and the whiskers show 5th to 95th percentiles. (**D**) UMAP representation of S100A12 expression in PBMCs from all samples analyzed by CITE-seq. (**E** and **F**) Correlation (Cor) analysis of S100A12 expression in cells from myeloid and dendritic cell clusters (C MONO_1, NC MONO, CDC2, PDC, C MONO_IFN, C MONO_2, and C MONO_3) with EN-RAGE levels in plasma (E) or HLA-DPA1 expression in the same clusters (F) (*n* = 5 healthy and 7 COVID-19 subjects). The statistical significance between the groups in (B) and (C) was determined by two-sided Mann-Whitney rank-sum test; *P < 0.05; **P < 0.01; ***P < 0.001.

In addition to an enhanced ISG signature, the CITE-seq analysis revealed a significant decrease in the expression of genes involved in the antigen-presentation pathways in myeloid cells (Fig. 4C and fig. S13). Consistent with this, we observed a reduction in the expression of the proteins CD86 and human leukocyte antigen class DR (HLA-DR) on monocytes and mDCs of COVID-19 patients, which was most pronounced in subjects with severe COVID-19 infection (Fig. 5A and fig. S16A). HLA-DR is an important mediator of antigen presentation and is crucial for the induction of T helper cell responses. Using the phospho-CyTOF data from both the Atlanta and Hong Kong cohorts, we confirmed the reduced expression of HLA-DR on monocytes and mDCs in patients with severe COVID-19 disease (Fig. 5B). By contrast, S100A12, the gene encoding EN-RAGE, was substantially increased in the PBMCs of COVID-19 patients, whereas the expression of genes encoding other proinflammatory cytokines was either absent or unchanged compared with healthy controls (Fig. 5C and fig. S16B). Notably, the S100A12 expression was highly restricted to monocyte clusters (Fig. 5D) and showed a significant correlation with EN-RAGE protein levels in plasma measured by Olink (Fig. 5E). Finally, we examined whether there is an association between HLA-DR and S100A12 expression in our dataset, and we found a strong inverse correlation between S100A12 gene expression and the genes encoding the antigen presentation machinery (HLA-DPA1, HLA-DPB1, HLA-DR, and CD74) (Fig. 5F and fig. S17). Notably, the receptor for S100A12, AGER (RAGE), was expressed sparsely in PBMCs (fig. S18), which suggests that the target of EN-RAGE action was likely to be elsewhere—perhaps the lung, where RAGE is known to be expressed in type I alveolar epithelial cells and mediate inflammation (*24*).

Taken together, CITE-seq analysis of PBMCs in COVID-19 patients revealed the following mechanistic insights: (i) a lack of expression of genes encoding type I IFN and proinflammatory cytokines in PBMCs, which was consistent with the mass cytometry (Fig. 1C) and functional data (Fig. 2); (ii) an early but transient wave of ISG expression, which was entirely consistent with analysis of RNA-seq from bulk PBMCs (Fig. 4G and fig. S15A) and strongly correlated with an early burst of plasma IFN-α (Fig. 4F), likely of lung origin (*17*); and (iii) the impaired expression of HLA-DR and CD86 but enhanced expression of S100A12 in myeloid cells, which was consistent with the mass cytometry (Fig. 5B), Olink (Fig. 3), and ELISA (fig. S7) data, and is a phenotype reminiscent of myeloid-derived suppressor cells described previously (*25*).

## Severe COVID-19 infection is associated with the systemic release of bacterial products

**Fig. 6:**
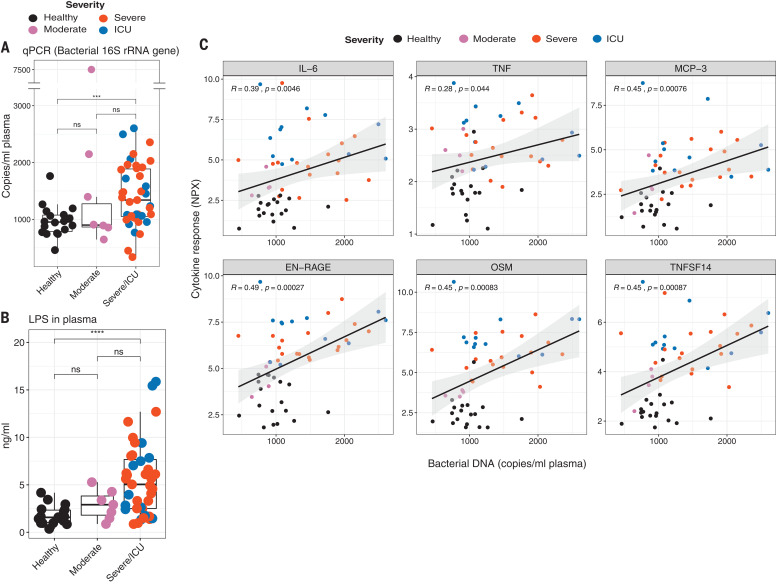
Systemic release of microbial products in severe COVID-19 infection. (**A** and **B**) Box plots showing bacterial 16*S* rRNA gene (A) and LPS (B) measured in the plasma of healthy or infected individuals. qPCR, quantitative PCR. (**C**) Spearman’s correlation between cytokines and bacterial DNA measured in plasma. Each dot represents a sample (*n* = 18 and 51 for healthy and infected, respectively). The colors of the dots indicate the severity of clinical disease, as shown in the legends. The boxes show median, upper, and lower quartiles in the box plots. The whiskers show 5th to 95th percentiles. The differences between the groups were measured by Mann-Whitney rank sum test; ***P < 0.001; ****P < 0.0001. NPX, normalized protein expression units; *R*, correlation coefficient.

The increased levels of proinflammatory mediators in the plasma—including IL-6, TNF, TNFSF14, EN-RAGE, and OSM (Fig. 3)—coupled with suppressed innate immune responses in blood monocytes and DCs (Fig. 2 and fig. S5) suggested a sepsis-like clinical condition (*26*, *27*). In this context, it has been previously suggested that proinflammatory cytokines and bacterial products in the plasma may play pathogenic roles in sepsis, and the combination of these factors could be important in determining patient survival (*28*, *29*). Therefore, to determine whether a similar mechanism could be at play in patients with severe COVID-19, we measured bacterial DNA and lipopolysaccharide (LPS) in the plasma. Notably, the plasma of severe and ICU patients had significantly higher levels of bacterial DNA, as measured by PCR quantitation of bacterial 16*S* ribosomal RNA (rRNA) gene product, and of LPS, as measured by a TLR4-based reporter assay (Fig. 6, A and B). Furthermore, there was a significant correlation between bacterial DNA or LPS and the plasma levels of the inflammatory mediators IL-6, TNF, MCP-3, EN-RAGE, TNFSF14, and OSM (Fig. 6C and fig. S19). These results suggest that the enhanced cytokine release may in part be caused by increased bacterial products in the lung or in other tissues.

## Discussion

We used a systems biology approach to determine host immune responses to COVID-19. Mass cytometry analysis of peripheral blood leukocytes from two independent cohorts revealed several common features of immune responses induced upon SARS-CoV-2 infection. There was a notable and protracted increase in the frequencies of plasmablasts and effector CD8 T cells in the peripheral blood, consistent with recent studies (*6*, *8*, *14*). Notably, the effector T cells continued to increase up to day 40 after symptom onset. Studies have shown that SARS-CoV-2 infection induces exhaustion and apoptosis in T cells (*30*, *31*). Whether the continuing effector CD8 T cell response reflects continuous exposure to antigen and whether the cells are exhausted will require further investigation.

In contrast to robust activation of B and T cells, we observed a significant decrease in the frequency of pDCs. Furthermore, mTOR signaling in pDCs was reduced significantly in COVID-19–infected individuals, as measured by decreased pS6 signaling by mass cytometry. These results suggest that pDCs, the primary producers of type I IFNs, are impaired in COVID-19 infection, which is consistent with studies in SARS-CoV infection (*32*). To determine whether the reduced mTOR signaling in pDCs resulted in impairment of type I IFN production, we stimulated cells in vitro with TLR ligands. Our results demonstrate that pDCs from COVID-19–infected patients are functionally impaired in their capacity to produce IFN-α in response to TLR stimulation. Taken together, these data suggest that COVID-19 causes an impaired type I IFN response in the periphery. Administration of type I IFN has been proposed as a strategy for COVID-19 intervention (*33*); however, it must be noted that type I IFN signaling has been shown to elevate angiotensin-converting enzyme 2 (ACE2) expression (*34*) in lung cells, which can potentially lead to enhanced infection.

In addition to the impaired IFN-α production by pDCs, there was a marked diminution of the proinflammatory cytokines IL-6, TNF-α, and IL-1β produced by monocytes and mDCs upon TLR stimulation (Fig. 2B). This was consistent with the lack of or diminished expression of the genes encoding IL-6 and TNF in the CITE-seq analysis (Fig. 5C). These results suggest an impaired innate response in blood leukocytes of patients with COVID-19. This concept was further supported by the CyTOF and flow cytometry data that showed decreased HLA-DR and CD86 expression, respectively, in myeloid cells (Fig. 5, D and E, and fig. S16). To obtain deeper insight into the mechanisms of host immunity to SARS-CoV-2, we performed CITE-seq single-cell RNA-seq and bulk RNA-seq analysis in COVID-19 patients at various stages of clinical severity. Our data demonstrate that SARS-CoV-2 infection results in an early wave of IFN-α in the circulation that induces an ISG signature. Although the ISG signature shows a strong temporal dependence in our datasets, we also find that the ISG signature is strongly induced in patients with moderate COVID-19 infection (Fig. 4G). Consistent with this, Hadjadj *et al*. (*5*) have reported an enhanced expression of ISGs in patients with moderate disease compared with those with severe or critical disease. Taken together, these data suggest that SARS-CoV-2 infection induces an early, transient type I IFN production in the lungs that induces ISGs in the peripheral blood, primarily in patients with mild or moderate disease. Additionally, we observed reduced expression of genes encoding proinflammatory cytokines, as well as HLA-DR expression in myeloid cells, which was consistent with the CyTOF and flow cytometry data showing reduced HLA-DR and CD86 expression, respectively, in myeloid cells.

Our multiplex analysis of plasma cytokines revealed enhanced levels of several proinflammatory cytokines, as has been observed previously (*35*), and revealed a strong association of the inflammatory mediators EN-RAGE, TNFSF14, and OSM with the clinical severity of the disease. Notably, the expression of genes encoding both TNFSF14 and OSM were down-regulated in the PBMCs from COVID-19 patients with severe disease in the analysis of CITE-seq data (Fig. 5C), which suggests a tissue origin for these cytokines. The gene encoding EN-RAGE, however, was expressed at high levels in blood myeloid cells in patients with severe COVID-19 (Fig. 5, C to F) (although it is also possible that EN-RAGE is expressed in the lungs too). Of note, these three cytokines have been associated with lung inflammatory diseases. In particular, EN-RAGE has been shown to be expressed by CD14^+^ HLA-DR^lo^ cells, the myeloid-derived suppressor cells, and it is a marker of inflammation in severe sepsis (*21*, *25*, *36*). Additionally, its receptor, RAGE, is highly expressed in type I alveolar cells in the lung (*24*). Notably, we observed that the classical monocytes and myeloid cells from severe COVID-19 patients in the single-cell RNA-seq data expressed high levels of S100A12, the gene encoding EN-RAGE, but not the typical inflammatory molecules IL-6 and TNF-α. These data suggest that the proinflammatory cytokines observed in plasma likely originate from the cells in lung tissue rather than from peripheral blood cells. Taken together with the mass cytometry data, the plasma cytokine data may be utilized to construct an immunological profile that discriminates between severe versus moderate COVID-19 disease (fig. S20).

These results suggest that SARS-CoV-2 infection results in a spatial dichotomy in the innate immune response, characterized by suppression of peripheral innate immunity in the face of proinflammatory responses that have been reported in the lungs (*37*). Furthermore, there is a temporal shift in the cytokine response from an early but transient type I IFN response to a proinflammatory response during the later and more severe stages, which is similar to that observed with other diseases such as influenza (*38*). Notably, there were enhanced levels of bacterial DNA and LPS in the plasma, which were positively correlated with the plasma levels of EN-RAGE, TNFSF14, OSM, and IL-6, which suggests a role for bacterial products—perhaps of lung origin—in augmenting the production of inflammatory cytokines in severe COVID-19. The biological consequence of the impaired innate response in peripheral blood is unknown but may reflect a homeostatic mechanism to prevent rampant systemic hyperactivation, in the face of tissue inflammation. Finally, these results highlight molecules such as EN-RAGE or TNFSF14, and their receptors, which could represent attractive therapeutic targets against COVID-19.

## Supplementary Materials

science.sciencemag.org/content/369/6508/1210/suppl/DC1 Materials and Methods Figs. S1 to S21 Tables S1 to S4 References (*39*–*43*) MDAR Reproducibility Checklist

## Appendix

View/request a protocol for this paper from *Bio-protocol*.
